# Phyto-Therapeutic and Nanomedicinal Approaches to Cure Alzheimer’s Disease: Present Status and Future Opportunities

**DOI:** 10.3389/fnagi.2018.00284

**Published:** 2018-10-23

**Authors:** Muhammad Ovais, Nashmia Zia, Irshad Ahmad, Ali Talha Khalil, Abida Raza, Muhammad Ayaz, Abdul Sadiq, Farhat Ullah, Zabta Khan Shinwari

**Affiliations:** ^1^Department of Biotechnology, Faculty of Biological Sciences, Quaid-i-Azam University, Islamabad, Pakistan; ^2^National Institute of Lasers and Optronics, Pakistan Atomic Energy Commission, Islamabad, Pakistan; ^3^CAS Key Laboratory for Biomedical Effects of Nanomaterials and Nanosafety, CAS Center for Excellence in Nanoscience, National Center for Nanoscience and Technology, Beijing, China; ^4^Department of Pharmacy, University of Peshawar, Peshawar, Pakistan; ^5^Department of Life Sciences, King Fahd University of Petroleum and Minerals, Dhahran, Saudi Arabia; ^6^Department of Eastern Medicine and Surgery, Qarshi University, Lahore, Pakistan; ^7^Department of Pharmacy, University of Malakand, Chakdara, Pakistan; ^8^Department of Life Sciences and Chemistry, Faculty of Health, Jacobs University Bremen, Bremen, Germany; ^9^Pakistan Academy of Sciences, Islamabad, Pakistan

**Keywords:** Alzheimer’s disease, nanotechnology, medicinal plants, green synthesis, theranostic NPs

## Abstract

Alzheimer’s disease (AD) is characterized by cognitive inability manifested due to the accumulation of β-amyloid, formation of hyper phosphorylated neurofibrillary tangles, and a malfunctioned cholinergic system. The degeneration integrity of the neuronal network can appear long after the onset of the disease. Nanotechnology-based interventions have opened an exciting area via theranostics of AD in terms of tailored nanomedicine, which are able to target and deliver drugs across the blood–brain barrier (BBB). The exciting interface existing between medicinal plants and nanotechnology is an emerging marvel in medicine, which has delivered promising results in the treatment of AD. In order to assess the potential applications of the medicinal plants, their derived components, and various nanomedicinal approaches, a review of literature was deemed as necessary. In the present review, numerous phytochemicals and various feats in nanomedicine for the treatment of AD have been discussed mechanistically for the first time. Furthermore, recent trends in nanotechnology such as green synthesis of metal nanoparticles with reference to the treatment of AD have been elaborated. Foreseeing the recent progress, we hope that the interface of medicinal plants and nanotechnology will lead to highly effective theranostic strategies for the treatment of AD in the near future.

## Introduction

Alzheimer’s disease (AD) is a chronic neurodegenerative disorder characterized by memory loss, impaired cognitive function, and behavioral instability, predominantly among the elderly population (Minati et al., 2009). In the brain of AD patients, the excessive production of proteins [β-amyloid (Aβ) and hyperphosphorylated tau] leads to the loss of synaptic connections and neuronal impairment in the hippocampus and cerebral cortex. The aforementioned events thus, lead to the degenerative diseases like AD and dementia (Selkoe, 2001). The complex pathophysiological aspects of AD brain have been extensively studied; however, an effective medicine is still unavailable for the complete treatment of AD (Ayaz et al., 2016). In clinical trials, several claimed potential anti-Alzheimer drugs failed to prove their effectiveness (Cummings et al., 2016). In order to combat the situation, alternative and cutting edge approaches like nanomedicine have been proposed as a potential treatment modality. In this review paper, emerging phyto-therapeutic and nanomedicinal approaches for treating AD are discussed. In addition, recent trends in emerging theranostics for AD using NPs delivery across the blood–brain barrier (BBB) and their mechanism of action are elaborated.

### Prevalence and Etiology of AD

The AD is one of the major causes of dementia in an estimated 60–80% of cases. According to the Alzheimer’s Association Report 2017, about 5.5 million individuals have AD (Alzheimer’s and Association, 2017). Early clinical symptoms of AD patients include impairment in memory, e.g., current conversations, names or events, while later symptoms comprises poor communication, misperception, confusion, misjudgment, changed attitude, and finally, difficulty in speaking, swallowing, and walking (Khachaturian, 1985). In the United States, AD was reported in 4.7 million individuals aged 65 years or older during 2010, which is estimated to reach 13.8 million cases by 2050. Similarly, a considerable number of deaths were observed among older AD patients. In 2010, ∼600,000 deaths occurred among people with AD at the age of 65 years or older, which is expected to reach 1.6 million by 2050 (Hebert et al., 2013).

The Aβ and tau proteins are the key factors to be considered in AD treatment. Currently, researchers are trying to develop drugs that can minimize amyloid synthesis/accumulation and tau aggregation/phosphorylation. The AD is associated with multiple factors of unknown etiology, and various cases can be observed with mutations in different genes, such as in presenilins (PS1 and PS2) and Aβ precursor protein (APP) (Bird, 2008). The apolipoprotein E (APOE) genes are also considered to be a risk factor for AD. Similarly, different proteins like APOE, APP, β amyloid cleaving enzyme (BACE), presenilin 1 and 2 (PS1 and PS2), and secretases (BACE1) are the key factors in the pathogenesis of AD (Kim et al., 2009). Therefore, researchers have focused on the synthesis of novel inhibitors for PS1, BACE, and BACE1 for the possible treatment of AD. Investigators are also trying to determine the efficacy of cholinesterase (ChE) inhibitors in the brain (Ayaz et al., 2017b; Ovais et al., 2018b). The appliance of new-generation acetyl and butyrylcholinesterase (AChE/BChE) inhibitors has been tested in the clinical trials for AD (Giacobini, 2004). Additional approaches such as hormone therapy and use of antioxidants, cholesterol-lowering mediators, anti-inflammatory substances, and vaccinations have also been studied for the treatment of AD (Yiannopoulou and Papageorgiou, 2013).

### Role of the BBB in AD

The BBB is a delicate membrane synthesized by the endothelial cells, which makes up the inside layer of cerebral microvessels. The BBB controls the entrance of plasma components, red blood cells, and leukocytes into the central nervous system (CNS), while exporting the neurotoxic molecules from the brain to the blood. Two additional sites were found in the CNS, which make up a barrier between the blood and cerebrospinal fluid (CSF), e.g., the arachnoid epithelium constituting the central layer of the meninges and choroid plexus epithelium. These distinctive BBB membranes encompass a group of physical, transport, and metabolic barriers that separate the neural environment from the blood (Abbott et al., 2010). The integrity of the BBB is controlled by the brain microvessel endothelial cells (BMECs) having distinctive luminal (apical) and abluminal (basolateral) membrane compartments (Zlokovic, 2011). They are an essential part of the neurovascular unit (NVU) in conjunction with pericytes, vascular smooth muscle cells from the vessel wall, neurons, and glial cells. Besides maintaining the chemical composition of the brain interstitial fluid, the NVU regulates BBB permeability and cerebral blood flow, which is necessary to maintain useful neuronal circuits (Daneman and Prat, 2015). Impairment in the BBB and NVU has severe pathological characteristics. These include outflow of circulating materials from the plasma into the CNS, changes in transporters that lead to poor nutrient supply and addition of neurotoxins in the CNS, and changes in the expression or secretion of proteins by NVU cells responsible for inflammation, oxidative stress, and neuronal damage. Recently, it has also been reported that the abnormal NVU cells result in different CNS pathologies such as dementia, neurodegeneration, and cognitive failure in AD (Erickson and Banks, 2013). Previously, the CNS was thought of as an immune-privileged organ, missing a lymphatic system and protected from the peripheral blood cells by the BBB. Conversely, the phenomenon is clear now that the BBB has the ability to respond to soluble factors and plasma proteins and interconnect with the peripheral immune system cells, thus forming neuroimmune system interactions, validating the concept that neuroinflammation leads to AD pathology (Heneka et al., 2015). Hence, the brain cannot be considered as an immune-privileged organ, and abnormal NVUs have to be withheld for peripheral immune cells and circulating soluble molecules that mediate immune responses (Zenaro et al., 2015).

### Alternative Therapy Using Medicinal Plants

Due to severe side effects of current therapies, alternative therapies such as use of medicinal plants for the treatment of AD and other diseases are a vital focus of researchers (Ali M. et al., 2017; Ayaz et al., 2017a; Sadiq et al., 2018). The medicinal plants have been reported to enhance the memory and learning process that normally decline with AD (Ahmad et al., 2016; Ayaz et al., 2017b; Zohra et al., 2018). From the current literature, we have summarized potential anti-Alzheimer compounds isolated from various medicinal plants (Table 1). Currently, several studies report on the phytochemicals that have been clinically proven with significant anti-AD potentials. Some of these bioactive compounds and their mechanisms of action against AD have been discussed in the following sections.

**Table 1:** The major medicinal plants with potential bioactive compounds and their mechanisms of action against AD.

Plant	Part used	Mechanism of action	Reference
*Withania somnifera*	Withanolides and withanamides	- Effectively inhibits β-site amyloid precursor protein cleaving enzyme (BACE1) and acetylcholinesterase (AChE).	Mahrous et al., 2017
		- Bind to the active motif of β-amyloid (25–35), protect the rat neuronal cells (PC-12) from β-amyloid induced cell damage and prevent the fibril formation.	Jayaprakasam et al., 2010
*Curcuma longa*	Curcumin	- Effectively inhibits Aβ oligomer and fibril formation in Tg2576 mice brain.	Yang et al., 2005
		- Reducing the astrocytic marker GFAP, Aβ, and plaque burden in Alzheimer transgenic APPSw mouse model (Tg2576).	Lim et al., 2001
*Convolvulus pluricaulis*	Convolvine and convolamine	- Reduce AD biomarkers (AβPP, tau, and Aβ) in Naive male Wistar rats.	Bihaqi et al., 2012
		- CNS depressant, neurodegenerative, and increases learning abilities.	Agarwa et al., 2014
		- Increased AChE activity in the hippocampal CA1 and CA3 regions associated with learning and memory.	Dubey et al., 1994
*Centella asiatica*	Asiatic acid asiaticoside	- Used for rejuvenating the neuronal cells, increasing intelligence, longevity, and memory.	Shinomol and Bharath, 2011 Dhanasekaran et al., 2009
		- Inhibits β-amyloid level in the brains of PSAPP mice.	
*Celastrus paniculatus*	Celapanin and celapanigin	- Increases cholinergic activity and improves memory performance by increasing ACh level in rat brain.	Bhanumathy et al., 2010
		- Prevents glutamine-induced neurotoxicity in embryonic rat forebrain neuronal cells.	Godkar et al., 2006
*Nardostachys jatamansi*	Valeranone nardosinone	- Improves learning and memory in mice.	Karkada et al., 2012
*Coriandrum sativum*	Coumarin	- Suppresses Aβ42, glial cell proliferation, and extracellular signal-regulated kinase activation.	Liu et al., 2016
		- Improves Aβ(1–42)-induced spatial memory impairment by attenuation of the oxidative stress in the rat hippocampus.	Liu et al., 2016
		- Improves memory deficits in rats induced by scopolamine and diazepam.	Mani and Parle, 2009
*Ficus carica*	Quercetin	- Induces neuronal bioactivity and is against oxidative stress-related AD.	Khojah and Edrada-Ebel, 2017
		- Improvement of cognitive and behavioral deficits.	Subash et al., 2016
*Ginkgo biloba*	Bilobalide ginkgolide	- Increases cell proliferation and neuroblast differentiation in mice.	Yoo et al., 2011
		- Effective in the treatment and prevention of AD.	Shi et al., 2010
*Coffea arabica*	Caffeine and chlorogenic acids	- Possesses neuroprotective effect in alleviating AD.	Shi et al., 2010
		- Modulates nicotinamide mononucleotide adenylyl transferase 2 levels in cortical neurons.	Ali Y.O. et al., 2017
*Glycyrrhiza glabra*	Glycyrrhizin	- Relives neuroinflammation and memory deficit prompted by systemic lipopolysaccharide treatment in mice.	Song et al., 2013
*Lepidium meyenii*	Choline	- Enhances learning and memory, while reduces lipid peroxidation and acetylcholinesterase in ovariectomized (OVX) mice.	Rubio et al., 2011
*Huperzia serrata*	Huperzine	- Promotes hippocampal neurogenesis *in vitro* and *in vivo* and activates MAPK/ERK signaling pathway in mice.	Ma et al., 2013
*Panax ginseng*	Ginsenoside	- Cholinergic and activates Rb1, Rg1-3, Re, and Rh2 effective in the treatment of AD.	Nie et al., 2017
		- Ginsenoside Rg1 improves behavioral abnormalities and modifies the hippocampal proteomic profile in transgenic AD mice.	
*Magnolia officinalis*	Magnolol and honokiol	- Recovering memory impairment induced by scopolamine, and its effect may be related to the ability of AChE inhibition.	Lee et al., 2009
		- Significantly decreases Aβ-induced cell death, neuroprotective, reduces ROS production, suppresses intracellular calcium, and inhibits caspase-3 activity.	Hoi et al., 2010
*Zingiber officinale*	Shogaol and gingerol	- Significantly improves learning and memory in rates.	Gharibi et al., 2013
		- Inhibits GSK-3β and helps to recover the SHSY-5Y cells from Aβ-42 toxicity.	Yerer et al., 2017
*Crocus sativus*	Crocin	- Reduces cognitive decline in AD patients.	Farokhnia et al., 2014
		- Inhibits Aβ and has a positive effect on cognitive function in AD.	Akhondzadeh et al., 2010
*Cissampelos pareira*	Benzylisoquinoline	- Inhibits acetylcholinesterase activity and enhances memory in mice.	Kulkarni et al., 2011
*Myristica fragrans*	Monoterpenoid	- Memory enhancement and inhibition of acetylcholinesterase activity.	Asgarpanah and Kazemivash, 2012
*Bacopa monnieri*	Bacoside and reserpine	- Inhibits acetylcholinesterase and β-amyloid level while activates choline acetyltransferase.	Aguiar and Borowski, 2013
		- Improved cognition, protects memory loss, and neurodegeneration	Shinomol and Bharath, 2011
*Camellia Sinensis*	Catechins	- Prevents cognitive impairment and protects brain by modulating the risk factors for AD.	Ho et al., 2013
		- Regulates BACE1 mRNA expression in SY5Y neuroblastoma cells.	Geiser et al., 2017
*Allium sativum*	S-allylcysteine	- Improves short-term recognition memory and relieves the neuroinflammation in Aβ-induced rats.	Nillert et al., 2017


#### Withanolides and Withanamides

*Withania somnifera* (WS), an essential medicinal plant, has been in use for the treatment of AD for the past 3000 years. The crude extract of this plant possesses withanamides A and C that can effectively bind to Aβ and inhibit fibril synthesis in the rat neuronal cells (PC-12) (Jayaprakasam et al., 2010; Ali et al., 2018). In a current study, withanolide S, isolated from different parts of WS, showed dual inhibitory activities against BACE1 and AChE (Mahrous et al., 2017; Puerta et al., 2017). In another study, computational tools were used to determine the bioactive compounds of WS against AD. The ligands (anaferine, anahygrine, cuscohygrine, and isaopelletierine) of WS were found to be neurologically active as agonists at the neuronal nicotinic acetylcholine receptors (nAChR) (Remya et al., 2016). Vareed et al. (2014) investigated the BBB permeability of withanamides that are present in *W.somnifera* fruit extract using animal models via high-performance liquid chromatography and quadrupole time of flight mass spectrometer system. Results of the study concluded that four withanamides significantly crossed BBB and their major peaks were identical both in brain tissue homogenates and plant crude extracts (Vareed et al., 2014).

#### Curcumin

Curcumin is the active ingredient isolated from *Curcuma longa*, carrying significant effects against AD. It has an antiamyloidogenic potential, which inhibits Aβ aggregation and downregulates BACE1 expression. It also possesses antitau function by inhibiting tau hyperphosphorylation and plays a key role in tau tangle clearance. In a randomized, placebo-controlled double-blinded study, Larry Baum et al. reported that curcumin exhibits neuroprotective effects in AD patients via several mechanisms, including the inhibition of Aβ aggregation, the inflammatory pathways, and free radical-induced neurodegeneration (Baum et al., 2008).

In another study, curcumin significantly reduced pro-inflammatory cytokines levels like interleukin (IL) 1β and oxidized proteins. Moreover, curcumin therapy considerably declined soluble and insoluble Aβ load in brain tissues and reduced astrocytic marker glial fibrillary acidic protein (GFAP) (Lim et al., 2001). The free radicals-scavenging, anti-amyloid, and anti-inflammatory effects of curcumin were also analyzed in transgenic and lipopolysaccharide (LPS) induced AD model. In both animal models, curcumin therapy reduced Aβ load and the chronic inflammation via inhibition of inflammatory cytokines (Begum et al., 2008). Furthermore, it can be used as a neuroprotective agent that promotes neuroplasticity and inhibits AChE activity (Serafini et al., 2017). Several other studies signify the potential use of curcumin in the management of neurodegenerative disorders like AD and its possible mechanism of action (Baum and Ng, 2004; Aggarwal and Sung, 2009).

In a study, Tsai et al. (2011) investigated the BBB penetration potentials of curcumin-loaded poly (lactic-co-glycolic acid) (PLGA) nanoparticles (NPs). Curcumin-brain concentrations and retention times were considerably increased in the cerebral cortex and hippocampus tissues of the animal brain (Tsai et al., 2011). Likewise, curcumin therapy is also reported to inhibit cerebral ischemia and reperfusion injury via prevention of BBB damage (Jiang et al., 2007). Yet another study on nano-curcumin preparations revealed significant improvements in cognitive function and reduction in amyloid load in transgenic animal model of AD (Cheng et al., 2013).

### Asiatic Acid and Asiaticoside

*Centella asiatica* is an important medicinal plant containing asiatic acid (AA) and asiaticoside. These neuroprotective phytochemicals have the potential to cross the BBB. Asiatic acid increases the viability of differentiated human neuroblastoma SH-SY5Y cells (Ternchoocheep et al., 2017). The neuroprotective effects of asiaticoside have been reported in primary cultured mouse cortical neurons exposed to glutamate-induced excitotoxicity invoked by *N*-methyl-D-aspartate (NMDA). It downregulates NMDA receptor subtype 2B (NMDAR2B or NR2B), which shows that asiaticoside can protect neurons from excitotoxicity induced by NMDA exposure via obstructing cell apoptosis and calcium overload (Qi et al., 2014). Asiatic acid isolated from *C. asiatica* is reported to mediate its neuroprotective effects via reduction of the BBB permeability and mitochondrial injury (Krishnamurthy et al., 2009).

#### Celapanin and Celapanigin

Phytochemical studies of *Celastrus paniculatus* (CP) show the presence of alkaloids like celapanin and celapanigin, which are extensively used as neuroprotective agents, memory enhancers, and in different CNS disorders. A recent study has proposed the protective effects of CP against 3-nitropropionic acid (3-NP)-induced neurotoxicity. It also prevents glutamine-induced neurotoxicity in embryonic rat forebrain neuronal cells. Glutamate receptor and/or NMDA receptor antagonists have shown neuroprotective effects against such damage (Godkar et al., 2006; Malik et al., 2017).

#### Valeranone and Nardosinone

*Nardostachys jatamansi*, a flowering herb that contains valeranone and nardosinone enhances the levels of key neurotransmitters such as gamma-aminobutyric acid (GABA), norepinephrine, dopamine, serotonin, and 5-hydroxyindoleacetic acid in the brain. Valeranone and nardosinone also inhibit inflammatory cytokines IL-1β, IL-6, and tumor necrosis factor alpha (TNF-α). Furthermore, they deactivate p38 mitogen-activated protein kinases (MAPKs) (Akram and Nawaz, 2017) https://www.ncbi.nlm.nih.gov/pubmed/26371857. It is also reported that nardosinone acts as an enhancer of nerve growth factor (NGF) (Zhang C. et al., 2014), dibutyryl cyclic AMP, and staurosporine-induced neurite outgrowth from PC12D cells by amplifying both the MAP kinase-dependent and -independent signaling pathways of dibutyryl-cAMP (dbcAMP) and staurosporine (Li et al., 2003). Nordosinone has been recently reported to significantly cross BBB and exhibit strong antidepressant activity (Jalali et al., 2018).

#### Coumarin

*Coriandrum sativum*, known as coriander, is an important medicinal plant, which contains coumarin as an active ingredient that has memory-enhancing and neuroprotective effects (Mani and Parle, 2009). *C. sativum* extract intake by AD model flies increases the reactive oxygen species (ROS) levels and number of glial cells. It also inhibits the epidermal growth factor receptor and induced phosphorylation of extracellular signal-regulated kinase (ERK) (Laribi et al., 2015). Currently, a series of 7-substituted coumarin derivatives have been designed and synthesized to display ChE and monoamine oxidase (MAO-B) inhibitory activities. The molecular modeling determined that the tested compounds are able to inhibit AChE-induced Aβ aggregation and, therefore, can be considered as promising multifunctional lead compounds for the treatment of AD patients (Joubert et al., 2017).

Among the natural coumarins, osthole is reported to exhibit neuroprotective effects via inhibition of oxidative stress-induced cerebral ischemia and inhibition of BBB disruption (Mao et al., 2011; Chen Z. et al., 2015). Several coumarin derivatives are reported to selectively inhibit MAO-B enzyme implicated in AD and prevent Aβ_1-42_ aggregation. Some of the test compounds cross BBB, chelate metal ions, and exhibit low toxicity in cell lines, which signify their use as potential anti-AD agents (Huang et al., 2015; Jameel et al., 2016).

#### Quercetin

Quercetin, an important polyphenol found in *Ficus carica*, has the ability to improve learning and memory when used in the treatment of AD (Biswas et al., 2015). It is considered as a multifunctional neuroprotective agent (Ossola et al., 2009). Currently, the effect of oral administration of nanoencapsulated quercetin was determined in a mouse model of AD. It was observed that the oral administration of nanoencapsulated quercetin in zein NPQ reduced the cognition- and memory-impairment characteristics of senescence-accelerated prone mouse (SAMP8). These observations appeared to be related with a decreased expression of the hippocampal astrocyte marker GFAP (Puerta et al., 2017).

Quercetin is reported to significantly cross BBB (Faria et al., 2014), accumulate in the target brain tissues of animal models, and reduce the oxidative stress-mediated neurodegeneration (Ishisaka et al., 2011). Moreover, quercetin is also reported to exhibit neuroprotective effects via inhibition of NO excessive production and overexpression of iNOS and downregulation of pro-inflammatory gene expressions like TNF-α, COX-2, and IL-1βin zebrafish (Zhang et al., 2011). Nanoencapsulated quercetin is reported to inhibit ischemic reperfusion-mediated neuronal damage in animal models (Ghosh et al., 2013).

#### Bilobalide and Ginkgolide

*Ginkgo biloba* (GB) contains very important constituents such as bilobalide and ginkgolide. They have been extensively studied for their CNS effects (Maclennan et al., 2002) and can be used for the possible treatment of AD patients (Ahlemeyer and Krieglstein, 2003; Shi et al., 2010). The dietary treatment of mice with GB extract upregulates the expression of genes encoding neuronal tyrosine/threonine phosphatase 1 and microtubule-associated tau in the cerebral cortex. The GB extract can also upregulate two mitochondrial DNA-encoded genes, subunit III of cytochrome-c oxidase and NADH dehydrogenase, subunit 1 (ND1), indicating a fundamental mechanism that may underlie bilobalide-induced neuroprotection and its role in cognitive impairment (DeFeudis, 2002). Recently, it has also been reported that the ectopic expression of APE1 enables neuronal cells to overcome the oxidative damage caused by Aβ_25-35_. Moreover, ginkgolide has modified the mitochondrial OXPHOS against Aβ_25-35_-induced oxidative stress and regulated the reactive oxygen and/or nitrogen species (ROS/RNS) level in the existence of ectopic APE1. This study portrays an alternative approach in the therapeutic potential for AD by harnessing the synergistic neuroprotective roles of apurinic/apyrimidinic endonuclease 1 (APE1) and ginkgolide (Kaur et al., 2015).

Ginkgolides A, B, C, J, and bilobalide isolated from *G. biloba* significantly cross BBB using *in vitro* and *in vivo* permeability models and inhibit oxidative stress-induced cognitive dysfunctions (Sharma et al., 2000; Madgula et al., 2010). In another brain permeability study, ginkgolide B passed BBB and inhibited reperfusion-induced ischemic brain damage (Fang et al., 2010; Lang et al., 2010).

#### Caffeine and Chlorogenic Acids

*Coffea arabica* is well known for the coffee, which is a common drink linked to a number of health benefits, including its key role to intervene AD. Different coffee varieties contain caffeine and chlorogenic acids in different concentrations, which possess neuroprotective effects observed in various *in vitro* and *in vivo* studies (Rebai et al., 2017). Recently, nicotinamide mononucleotide adenylyl transferase 2 (NMNAT2) was reported as a key neuronal factor that delivers strong neuroprotection in numerous preclinical models of neurological disorders. Systemically administered caffeine was identified as a positive modulator that restores NMNAT2 expression in tauopathy mouse model (rTg4510) to normal levels (Ali Y.O. et al., 2017). Caffeine and 5-caffeoylquinic acid (chlorogenic acid)-enriched coffee extensively crosses BBB and exhibits numerous CNS effects including neurotropic, anti-AD, and neuroprotective properties (Ito et al., 2008; Chu et al., 2009; Kwon et al., 2010; Cropley et al., 2012; Esquivel and Jiménez, 2012; Oboh et al., 2013).

#### Glycyrrhizin

Glycyrrhizin (GRZ) is a triterpenoid saponin, majorly found in *Glycyrrhiza glabra*. The GRZ exhibited spatial memory-enhancing and ameliorating effects on cognitive impairment induced by Aβ injection into the hippocampus *in vitro* and *in vivo* (Grzybowska et al., 1998; Chen et al., 2017). Currently, it is being reported that GRZ effectively reduced neuroinflammation and ameliorated the memory deficits induced by systemic LPS treatment. The effects of GRZ were found to be mediated through inhibition of pro-inflammatory cytokines TNF-α, IL-1β, and microglial activation in the brain tissues. This study supports that GRZ may be a putative therapeutic drug for neurodegenerative diseases that are associated with cognitive deficits such as in AD (Song et al., 2013).

The BBB permeability of 18β-glycyrrhetinic acid, an active metabolite of GRZ, was reported by Tabuchi et al. (2012) using *in vitro* and *in vivo* models. Study results revealed that the test compound, after oral administration, significantly crossed the BBB in both the *in vitro* BBB permeability model as well as animal models (Tabuchi et al., 2012). Oral pretreatment with GRZ is reported to thwart surgery-mediated cognitive dysfunctions via inhibition of neuroinflammation and AD symptoms (Chen et al., 2017).

#### Choline

Choline is required for the production of acetylcholine, an important neurotransmitter for memory, brain, and nervous system functions. It also plays important roles in modulating gene expression, cell membrane signaling, lipid transport and metabolism, and early brain development. Nutritional intake of choline in AD patients can influence cognitive functions via an effect on phosphatidylcholine (PC)-containing eicosapentaenoic and docosahexaenoic acids and polyunsaturated species of PC, levels of which are reduced in brain that is linked with higher memory performance (Blusztajn et al., 2017). In a current study, perinatal choline supplementation was reported to reduce amyloidosis and increase choline acetyltransferase expression in the hippocampus of APPswePS1dE9 (APP/PS1) AD mice. The data propose that dietary intake of choline during fetal development and early postnatal life can establish a preemptive approach for AD (Mellott et al., 2017).

#### Huperzine

Huperzine A (HupA) is a lycopodium alkaloid found in *Huperzia serrata*. The HupA was reported as an effective cholinergic agent. It is also effective in acute seizures as tested in a rat model. Therefore, HupA is a safe drug for AD patients who show both mnemonic and epileptic symptoms (Rafii et al., 2011). Moreover, it has also been reported that HupA can penetrate the BBB due to its interaction with efflux transmembrane transporters (ABCB1 and ABCG2). In transgenic animal models, the brain-to-plasma concentration ratio of Huperzine A was significantly increased as compared with the wild type mice due to ABCB1 that played a predominant role in the efflux of Huperzine A across BBB (Li et al., 2017).

#### Ginsenoside

Ginsenosides Rg1 (GRg1) and Rb1 (GRb1) are found to be the active ingredients of ginseng, which exhibits potential efficacy in prevention and treatment of CNS disorders and neurodegenerative diseases like AD. Currently, Rb1 and baicalin have been reported to promote proliferation and differentiation of endogenous neural stem cells (NSCs) in AD rat models. It also shows a decreased nuclear pyknosis and pyramidal cell defects in hippocampus of rats by increasing the expression of nestin, GFAP, and neuron specific-enolase (NSE) proteins and thereby improves cognitive function in AD rats (Zhao et al., 2018). Recently, the neuroprotective effects and its transport across BBB was reported by Rui et al. (2009) using *in vitro* and *in vivo* rat models. Ginsenoside Rg1 exhibited significant neuroprotective potentials, whereas, its passage across BBB was extremely poor. Ginsenoside Rg1 ameliorates cerebral ischemia via downregulation of protease-activated receptor 1 (PAR-1) expression (Xie et al., 2015) and offers protection against BBB disruption in brain injury model (Zhou et al., 2014; Chen W. et al., 2015).

#### Magnolol and Honokiol

Magnolol and honokiol are the bioactive compounds found in *Magnolia officinalis*. They are neuroprotective and decrease ROS production, intracellular calcium and caspase-3 activities. Therefore, these phytochemicals or their analogs may be explored as therapeutic drugs for AD (Hoi et al., 2010). Honokiol was reported as to reduce AβO-induced hippocampal neuronal apoptosis, ROS production, and loss of mitochondrial membrane potential in a dose-dependent manner. Moreover, honokiol inhibited ABO-mediated NF-κB activation as well as inhibited the up regulation of amyloid precursor protein (APP) and beta secretase enzyme (BACE1). Therefore, honokiol may be a potential candidate in AD therapy (Wang et al., 2017). Honokiol is reported to cross BBB and effectively target CNS disorders like gliosarcoma (Wang et al., 2011) and cerebral ischemia via inhibition of NF-κB (Zhang et al., 2013) and offer protection against brain ischemic-reperfusion injury via disruption of PSD95–nNOS interactions (Hu et al., 2013).

#### Shogaol and Gingerol

*Zingiber officinale* contains two major therapeutic agents, shogaol and gingerol, that could recover the SHSY-5Y cells from Aβ_1-42_ oligomer and aggregate toxicity. Currently, 6-gingerol and 6-shogaol have been found as neuroprotective agents, which can inhibit the GSK-3β activity (Yerer et al., 2017). It was found that 6-shogaol influenced neuritogenic activity in PC-12 cells by activating the MAPK, extracellular signal-regulated kinase 1 and 2 (ERK1/2), phosphatidylinositol 3-kinase (PI3K), and protein kinase B (AKT) signaling pathways. Hence, 6-shogaol can act as a NGF mimicking agent, which can be used as a preventive therapeutic agent in neurodegenerative diseases (Seow et al., 2017).

#### Crocin

Crocin is a water-soluble carotenoid found in *Crocus sativus* and possesses the capability to improve learning and memory as well as protect brain cells. Recently, crocin has been reported for its considerable effects on Aβ and tau proteins. Crocin revealed multifunctional protective activities in the brain and could be a promising agent when applied as a supplement or drug for the treatment of AD (Finley and Gao, 2017). Crocin has been reported to improve locomotor activities in animal models (Karami et al., 2013), provide protection against oxidative stress and cerebral ischemia (Zheng et al., 2007), and maintain BBB integrity (Zhang et al., 2017).

#### Benzylisoquinoline

*Cissampelos pareira* contains benzylisoquinoline that exerts nootropic activity in AD. It is a memory-enhancing agent, inhibits AChE activity, and enhances memory in mice (Kulkarni et al., 2011). A novel series of benzylisoquinoline derivatives have also been evaluated. The results have shown that most of the compounds have significant inhibitory potentials against ChEs (James et al., 2016), human cholinesterases (h-ChEs), and self-induced Aβ aggregation (Xu et al., 2014).

#### Monoterpenoid

Monoterpenoids in *Lavandula luisieri* have been identified as potent inhibitors of β-secretase (BACE-1), an aspartic protease involved in the conversion of APP to Aβ in animal models. Currently, an active monoterpene, necrodane ketone, 2,3,4,4-tetramethyl-5-methylenecyclopent-2-enone was tested for its inhibitory mechanism against BACE-1. This monoterpene revealed a dose-dependent inhibition of BACE-1 in cellular and mouse models of AD (Videira et al., 2014). Recently, 80 types of aroma compounds including monoterpenes were screened for *in vitro* inhibitory activity at a concentration of 200 μM against recombinant human BACE1 (Marumoto et al., 2017).

#### Bacoside and Reserpine

*Bacopa monnieri*, known as a memory-enhancing herb, contains key therapeutic agents bacoside and reserpine. It promotes free radicals scavenger mechanisms and protects cells in the prefrontal cortex, hippocampus, and striatum against cytotoxicity and DNA damage implicated in AD. It protects the cholinergic neurons and reduces anticholinesterase activity comparable with donepezil, rivastigmine, and galantamine (Chaudhari et al., 2017). The mechanism of action of reserpine was determined in *Caenorhabditis elegans* where it alleviated the toxicity of Aβ. The afore-mentioned study was conducted to evaluate the ∼2.8 kb promoter region of FLP-11, driving the expression of green florescent protein, which can be a critical mediator of alleviation of Aβ toxicity through acetylcholine and reserpine (Saharia et al., 2016). The PLGA-loaded bacoside-A NPs are reported to effectively deliver the drug across BBB and mediate its neuropharmacological actions (Jose et al., 2014; Chaudhary and Bist, 2017).

#### Catechins

Catechins found in *Camellia sinensis* prevent cognitive impairment among the elderly. It provides protection to the brain and modulates the risk factors of AD (Preedy, 2012). Currently, the human-derived cell line (SH-SY5Y) was treated with native oligomers (epicatechin and theaflavin) that reduce Aβ-induced BACE1 expression. The data show that the antioxidant activity of catechins and theaflavins may be more essential in downregulating BACE1 mRNA expression than their capability to inhibit Aβ oligomerization (Geiser et al., 2017). Catechins can cross the BBB and mediate various neuroprotective effects (Faria et al., 2011; Wu et al., 2012).

#### *S*-allylcysteine

Garlic extract (GE) contains *S*-allylcysteine, which has been reported for multiple biological activities, including anti-inflammatory effect and its influence on Aβ_1-42_-induced cognitive dysfunction and neuroinflammation (Kodera et al., 2002; Pérez-Severiano et al., 2004). The GE was found to be useful by improving the short-term recognition memory and relieve the neuroinflammation in Aβ-induced adult male Wister rats (Nillert et al., 2017). Recently, a research group has reviewed different antioxidant mechanisms (scavenging of free radicals and prooxidant species, induction of antioxidant enzymes, activation of Nrf2 factor, inhibition of prooxidant enzymes, and chelating effects) involved in the protective actions of aged GE (AGE) and S-allyl-L-cysteine (SAC), thereby, emphasizing their potential use as therapeutic agents (Colín-González et al., 2012).

### Treating Alzheimer’s: A Triple Challenge

Treating brain diseases, in general, and AD, in particular, is a special challenge. First of all, the intricate and specific system that the brain possesses in the form of BBB analyzes and monitors the transport of every molecule that crosses the brain. It consists of endothelial cells, astrocytes, pericytes, and microglial cells (Johansson, 1990). The highly tight junctions between the endothelial cells act as a border patrol system, which analyze the molecules on the basis of their lipophilicity, size, surface charge, and hydrogen-bonding potential. Priority is given to small lipophilic molecules with size less than 500 Da and the entry of these molecules is restricted, which do not fall under the specific criteria of structural and physicochemical properties (Abbott et al., 2010). In addition, even if the molecule of choice does get entry across the BBB, there is a high chance of it being thrown out due to the number of efflux transporters such as the ATP-binding cassette family (P-glycoprotein ABCB1), the multidrug resistance-related proteins (MRPs, ABCC1, 2, and 5) and the breast cancer resistance protein (BCRP, ABCG2) present in the endothelium of the brain _ENREF_3 (Begley, 2004; Ayaz et al., 2017d).

In addition to the transport-related issues, limited knowledge of the basic pathology of AD is another challenge in more effective drug therapy (Sperling et al., 2013). Many mechanisms leading to the pathology have been proposed, and the disease is attributed to be the result of a number of overlapping phenomena such as inflammation, iron dysregulation, oxidative damage, cholesterol metabolism (Galimberti and Scarpini, 2010), and “amyloid plaque” hypothesis. Despite this, there is no FDA-approved drug or carrier system with the disease-modifying potential for AD (Casey et al., 2010). Moreover, the third and major limitation in the successful therapy of AD is the absence of tools for early detection of the disease. Biochemical alterations at the cellular level start around 10–20 years before the onset of cognitive decline (Beason-Held et al., 2013). It has been proposed that the failure of various therapeutic approaches may be attributed to the fact that until the time of appearance of clinical symptoms, irreversible damage has already been done.

### Currently Approved Drugs for Alzheimer’s and Their Shortcomings

Until now, the FDA has approved drugs only for the symptomatic treatment of AD. The five that have been approved target neurotransmitter imbalance at different stages of disease; they include AChE inhibitors; tacrine (1st generation), donepezil, rivastigmine, and galantamine (2nd generation) (Ayaz et al., 2015). One of the five approved drugs by the FDA for the symptomatic treatment of AD is an NMDA receptor blocker, memantine. The pharmacokinetic profile of drugs affecting acetylcholine and NMDA is given in Table 2.

**Table 2:** The pharmacokinetic profile of FDA-approved drugs for AD.

Name	Vdl	Log p	Molecular weight g/mol	Formulation (oral)		References
				Excretion [renal and hepatic]	T_1/2_	
Memantine	9 to 11 L/Kg	3.3	179.307 g/mol	148% of administered drug is excreted unchanged in urine	60–100 h	(1)
Rivastigmine	1.8 to 2.7 L/kg	2.3	250.342 g/mol	Renal clearance = 2.1–2.8 L/h	1.5 h	(2)
Donepezil	12 L/kg	3.6	379.5 g/mol	0.13 L/h/kg in urine	70 h	(3)
Galantamine	175 L	1.8	287.359 g/mol	300 mL/min [After IV. or oral administration] in urine	7h	(4)


*(1) Drug Bank. Memantine. 2018, September 29 Available from: https://www.drugbank.ca/drugs/DB01043.*

*(2) Drug Bank. Rivastigmine. 2018, September 29 Available from: https://www.drugbank.ca/drugs/DB00989.*

*(3) Drug Bank. Donepezil. 2018, September 29 Available from: https://www.drugbank.ca/drugs/DB00843.*

*(4) Drug Bnak. Galantamine. 2018, September 29 Available from: https://www.drugbank.ca/drugs/DB00674.*

Despite the beneficial effects of improvement in everyday life of patients, reduction in emotional impact on caregivers, and overall care cost, the large variations between the effectiveness and efficacy of these drugs make their cost-effectiveness controversial (Loveman et al., 2006). This large gap in the efficacy profile is majorly due to peripheral stimulation of cholinergic system that results in gastrointestinal tract (GIT)-related side effects. For instance, GIT-based adverse drug reactions (ADRs) were the major causes for trial discontinuation, with mean frequency of nausea being 11% for donepezil, 44% for rivastigmine, and 24% for galantamine across various trials of AChE inhibitors (AChEI) (Casey et al., 2010). Moreover, these drugs have shown poor long-term maintenance of the improvement of symptoms with studies showing that benefits of AChEI can be endured for up to 4 years with decline in the cognition below baseline levels after 1 year of therapy (Winblad and Jelic, 2004).

All these drawbacks can be attributed to the poor pharmacokinetic profile of these therapeutic moieties, including low bioavailability, volatility, oxidation, hydrolysis, limited transport across BBB, and tendency for drug–drug interactions (Mangialasche et al., 2010). As AD majorly affects the elderly, the comorbidities, polypharmacy, and non-compliance have a huge impact in decreasing the efficacy of the treatment regimen (Noetzli and Eap, 2013). Therefore, there is a major need for the development of multipurpose moieties with potent therapeutic potential against AD.

### Mechanistic Basis of the Nanoparticle-Based Treatment

The unknown pathology and increase in the elderly population point toward a growing need for the development of therapeutic molecules specific to AD etiology. Many researchers around the world have developed NPs aiming at a different molecular mechanism of AD, in the hope of pinpointing the basic cause or limiting factor (Nazem and Mansoori, 2011; Table 3). There are four major and basic points in the pathophysiology of AD that can be targeted by use of NPs. **(1)** Neurotransmitter modulators; (**2)** anti-amyloid beta immunotherapy; (**3)** metal stress reducers, and (**4)** inflammatory inhibitors.

**Table 3:** Phytochemicals used as drug delivery agents for synthesis of NPs in treatment of AD.

Phytochemical NPs	Mechanism of action	Reference
Curcumin-NPs	-Oral administration of nanocurcumin to Tg2576 AD model mice even at a low dose (23 mg/kg per week) resulted in significant improvements over placebo control in working and cue memory. The novel nanocurcumin has a great potential for AD therapy.	Cheng et al., 2013
	-Curcumin-encapsulated PLGA nanoparticles (Cur-PLGA-NPs) potently induce neural stem cells (NSCs) proliferation and neuronal differentiation *in vitro* and in the hippocampus and subventricular zone of adult rats, as compared to uncoated bulk curcumin.	Tiwari et al., 2013
Quercetin-NPs	-PLGA-functionalized quercetin (PLGA@QT) NPs showed negligible cells toxicity and inhibited Aβ_42_ fibrillation and reduced Aβ_42_-induced toxicity. Morris water maze and Novel object recognition tests showed an enhanced learning and memory of AD mice and proposed it as therapeutic drug for AD.	Sun et al., 2016
	-Quercetin nanoparticles (NQC) enhancement has shown increased efficacy and prolonged residence time in systemic circulation and increased bioavailability in rates. The NQC offers the potential clinical application in human neurodegenerative disease in future.	Palle and Neerati, 2017
Ginsenoside-NPs	-Poly (lactic-co-glycolic acid) (PLGA)-NPs encapsulating ginsenoside Rg3 and Thioflavin T(Aβ diagnostic) were examined for their neuroprotective effects. Key mechanisms were investigated for their neuroprotective effects and evaluated their ability to cross the BBB using an *in vitro* BBB model. The PLGA-Rg3 NPs offer an exciting new theranostic material capable of encapsulating natural nutraceuticals for the detection and treatment of AD.	Aalinkeel et al., 2018
	-Ginsenoside Rg1 nanoparticle has potential to penetrate the blood–brain barrier to improve the cerebral function of diabetic rats complicated with cerebral infarction.	Shen et al., 2017
Bacoside-NPs	-Bacoside-A-loaded PLGA nanoparticles surfaces were modified by coating with polysorbate 80 to facilitate the crossing of the blood–brain barrier (BBB). When compared to pure drug solutions (2.56 ± 1.23 μg/g tissue), an *in vivo* study using Wistar albino rats demonstrated higher brain concentrations of Bacoside-A (23.94 ± 1.74 μg/g tissue) suggesting a significant role of surface-coated nanoparticles on brain targeting.	Jose et al., 2014
	-Bacoside as a model drug was incorporated into solid lipid nanoparticles (SLNs) prepared from stearic acid using Tween 80 emulsifiers, and *in vitro* drug release study showed up to 84.68% drug release from SLNs.	Vitthal et al., 2013
Huperzine-NPs	-Huperzine A using lactoferrin-conjugated *N*-trimethylated chitosan surface-modified PLGA NPs (Lf-TMC NPs) were developed for intranasal delivery to the brain for the treatment of AD. After intranasal administration, Lf-TMC NPs facilitated the distribution of HupA in the brain, and the values of the drug targeting index in the mouse olfactory bulb, cerebrum (with hippocampus removal), cerebellum, and hippocampus were about 2.0, 1.6, 1.9, and 1.9, respectively.	Meng et al., 2018
Choline -NPs	-Choline transporter (ChT) was used to transport choline-derivate-modified NPs across the blood–brain barrier. These modified NPs exhibited higher permeability across the brain capillary endothelial cells (BCECs) monolayer *in vitro* and higher gene distribution and expression *in vivo*.	Li et al., 2011
Lectin -NPs	-Basic fibroblast growth factor (bFGF) was entrapped in nanoparticles conjugated with *Solanum tuberosum* lectin (STL), which selectively binds to *N*-acetylglucosamine on the nasal epithelial membrane for its brain delivery. The spatial learning and memory of AD rats in the STL-bFGF-NP group were significantly improved compared with the AD model group proposing it a good brain drug delivery system for peptide- and protein-based drugs.	Zhang C. et al., 2014


#### Neurotransmitter Modulators

It is known that presynaptic deficits of Ach contribute significantly toward cognitive dysfunctions and induction of memory impairment (Ullah et al., 2016). Interestingly, it is noted in animal models that degeneration of neurons in the basal forebrain and medial septum leads to substantial decline in Ach levels in the cerebral cortex and hippocampus, respectively. Moreover, the biochemical evaluation of brain from patients with AD also showed considerable deficits in the neocortical levels of AChE, an enzyme involved in the synthesis of Ach (Ayaz et al., 2014). The NPs capable of transporting the acetylcholine-modulating drugs across the BBB should restore the imbalance of the Ach in AD patients and should ameliorate the deterioration of memory and improve cognitive function. It was reported that rivastigmine-loaded lipid NPs (RL) have successfully normalized the cortical Ach level and subsequently improved the deterioration of spatial memory in animal models treated with AlCl_3_ (Ismail et al., 2013). In their study, rats that were coadministered with RL and AlCl_3_ showed normalization of BACE1 gene expression as compared with its 316% overexpression in rats treated with AlCl_3_ only. Furthermore, the histological evaluation showed that coadministration of RL prevented the formation of Aβ plaque and, hence, strengthens the argument that normalization of Ach is associated with an anti-Alzheimer effect.

#### Amyloid Beta-Targeted Immunotherapy

The Aβs were considered as therapeutic agents for active immunity after it was noted that transgenic AD mouse developed anti-Aβ antibodies when immunized with Aβ_42_. The antibody development resulted in improved cognition and decreased amyloid plaque number. The mechanism involves binding of anti-amyloid antibody to the amyloid plaque followed by the disaggregation of plaques and, hence, removal from the brain (Modi et al., 2010). Previously, external brain molecules like heparin and gelsolin were thought to trap or “sink-in” the Aβ and, hence, decrease its concentration in the brain (Fonseca-Santos et al., 2015). Both active and passive immunotherapy targeting Aβ forms the leading approach in the treatment of AD.

For active immunotherapy, Aβ subfragments were used in combination with NPs as their delivery across BBB is low. This approach was used by Songjiang and Lixiang (2009) to develop intramembranous Aβ fragment-loaded chitosan NPs for enhanced brain delivery. The ELISA data from the study showed that the formulation has significant potential to induce immunogenicity (Songjiang and Lixiang, 2009). Similarly, Agyare et al. (2008) developed chitosan polymeric NPs with tripolyphosphate (TPP) and loaded them with the polyamine-modified F(ab’) portion of anti-amyloid antibody (IgG4.1). The biodistribution studies in mice and transport study across bovine BMECs (BBMECs) showed that NPs were able to trancytose across BBMEC, and, hence, are proposed to target brain amyloid (Agyare et al., 2008).

In addition to active immunity with Aβ fragments, passive immunization is widely exploited for potential therapeutic regimen in AD, as indicated by several Aβ-specific antibodies being present in various phases of clinical trials (Table 4). However, very few studies have exploited antibodies in combination with NPs for the treatment of AD. Currently, the first synthetic study of Aβ_1-42_ monoclonal antibody-tagged polymeric NPs (125 nm) has been reported for the treatment of AD in transgenic (Tg) 2576 mice. The study showed that animals treated with mAb-tagged NPs exhibit significant reduction in the soluble Aβ peptide and its oligomer level in the brain and considerable increase of the Aβ levels in plasma. Moreover, the results demonstrated complete recovery of memory deficits in Tg AD mice, which further strengthens the fact that antibody-mediated treatment may provide the ultimate long-term and personalized therapeutic strategy for AD treatment (Carradori et al., 2018).

**Table 4:** Properties of some important antibodies and BACE inhibitors at different phases of trials.

Name	Company	Phase trial	Observations	Target site	Nature	Reference
Solanezumab	Lilly, Roche, Alzheimer’s Association	II/III	Slowing of cognitive and functional decline versus placebo	monomeric Ab	IgG1 Anti amyloid monoclonal antibody	Siemers et al., 2016
			Baseline to endpoint changes in levels of CSF total tau and p-tau did not differ between treatment groups			
Aducanumab	Biogen Inc.	Ib	Slowing in clinical decline	amyloid-β (Aβ) plaques, tau	Anti amyloid monoclonal antibody	Sevigny et al., 2016
			Dose-dependent decrease in amyloid beta			
CAD106	Novartis, Amgen, NIA, Alzheimer’s association	II	Sustained Aβ-IgG titers and prolonged time to decline were observed in extensions versus core studies	Ab1–6, active vaccine	Anti amyloid vaccine	Farlow et al., 2015
Verubecestat	Merck	II/III	No clinically beneficial effect	BACE1	BACE1 inhibitor	Kennedy et al., 2016
			Reduction in plasma, brain, and CSF concentration of Ab40, Ab42, and sAPPb			
Bapineuzumab	Janssen Alzheimer Immunotherapy and Pfizer	III	No improvement in clinical outcomes of patient	Amyloid beta	humanized anti-amyloid-beta monoclonal antibody	Salloway et al., 2014
			Reduction in CSF level of phospho-tau			
			May modify Ab, but no clinical benefit (limitation of study design)			
Gantenerumab	Roche, Lilly, Alzheimer’s Association	I	mAb targeting aggregated Ab	Aggregated Ab	Anti-amyloid mAb	Bohrmann et al., 2012
Crenezumab	Genentech	II/III	–	Soluble oligomer and fibrillar Ab	Anti-amyloid mAb	Cummings et al., 2016
AADvac1	Axon Neuroscience	I,II	–	Tau epitope	Anti-tau mAb	Cummings et al., 2016
ABBV-8E12	AbbVie	II	–	tau	Anti tau monoclonal anti body	Cummings et al., 2016
BAN2401	Eisai	II	–	N terminal protofibrils	Monoclonal anti body	Cummings et al., 2016
NewGam 10% IVIG	Sutter Health	II	–	Multiple forms of Aβ	Polyclonal antibody	Cummings et al., 2016
LY3314814	Lilly	II/III	Being studied for MCI to mild AD	BACE	BACE inhibitor	Cummings et al., 2016
CNP520	Novartis	II/III	Being studied for asymptomatic (homozygote APOE 𝜀4/𝜀4)	BACE	BACE inhibitor	Cummings et al., 2016


#### Metal Stress Modulators

It is evident that metabolism of zinc, cupric, and iron metal ions regulates Aβ toxicity, senile plaques (SPs), and neurofibrillary tangles (NFTs) formation. It has also been reported that SP and AB plaques are enriched with copper, iron, and zinc (James et al., 2016). In addition to promoting the conversion of Aβ to oligomers and fibrils and hyperphosphorylation of tau, these metal ions gets reduced when they undergo binding with Aβ, leading to generation of H_2_O_2_ – the main source of oxidative stress in Aβ toxicity (França et al., 2017).

Metal chelators loaded onto NPs can easily gain access to brain, bind to the metal particles, and leave the brain along with their conjugated metal ions and, hence, provide an easy means to reduce metal stress. Cui et al. (2005) reported the synthesis of Cu (I) chelator D-penicillamine conjugated ethanolamine-based NPs. The study showed successful uptake of NPs in the brain, and the NPs along with released penicillamine were able to resolubalize more than 40 % of Cu–Ab conjugates when treated with 0.1 N NaOH (Cui et al., 2005). Similarly, Liu et al. (2009) reported the syntheses of polystyrene-based – iron chelator conjugate (Nano-N2PY) and its ability to inhibit Aβ aggregation-mediated cytotoxicity in human cortical neuronal cells. These preliminary *in vitro* studies can provide a strong background for further *in vivo* evaluation of metal-conjugated NPs as AD therapeutics.

#### Anti-inflammatory Agents

It is established that oxidative stress related to Aβ toxicity results in mitochondrial damage, which leads to cell death and neuronal damage. Anti-inflammatory and antioxidant agents, such as CoQ_10_, have established a role in mitochondrial bioenergetics and reduction of oxidative stress secondary to Aβ toxicity (Garrido-Maraver et al., 2014). The CoQ_10_-loaded trimethylated chitosan (TMC)–PLGA-based NPs (TMC-coated D,L-lactide-co-glycolide) were synthesized, and it was investigated that the drug-loaded TMC–PLGA NP formulation was biocompatible, showed higher accumulation in the brain, and with great reduction in memory impairment, restoring it to almost normal level in comparison with CoQ_10_-loaded PLGA NPs without TMC. The formulation also helped decrease Aβ concentration and Aβ fibril formation; this can be attributed to the effect of Co Q_10_ on dissolution of SP (Garrido-Maraver et al., 2014).

#### The NPs as Anti-Alzheimer Moieties; Overcoming the Existing Barriers

Traditional drugs used for AD have low bioavailability, limited transport, and controversial long-term effects; therefore, a tremendous number of studies have reported on the use of nano-scale particles to overcome these limitations. There are different strategies that are employed to use NPs of various origins to improve pharmacokinetic profiles; **(i)** increase targeted delivery of drugs across BBB (Rocha, 2013), **(ii)** increase efficacy of already available approved agents, and **(iii)** provide a multifunctional platform that can help in theranostic applications.

The NPs by virtue of their small size, modifiable surface properties, and tunable morphology act as ideal candidates to overcome all the hurdles that a brain drug delivery (DDR) system needs to evade (Lockman et al., 2002). With the large loading capacity, NPs are good candidates for carrying increased amounts of drug across the BBB and increasing the brain-to-blood ratio of the already available drugs (Fazil et al., 2012).

Various strategies have been adopted to enhance the effect of these tiny cargos for better therapeutics.

(1) Lipophilic NPs like liposomes for enhanced permeation of hydrophilic drugs across BBB (Smith et al., 2010).
(2) Hydrophilic polymer-based NPs are employed to impart stealth character and act as a parenteral sustained drug-release system by prolonging the blood retention time (Wilson et al., 2010). Thus, by providing a higher concentration gradient between blood and brain, they facilitate the passive diffusion of drug across BBB (Masserini, 2013).
(3) Mucoadhesive polymeric NPs are used for enhanced nasal delivery of drugs through direct endocytosis-mediated uptake across BBB (Mistry et al., 2009).
(4) Targeted NPs with surface ligands for receptor-mediated transcytosis (Ulbrich et al., 2009).

### Conventional NPs

Nanoparticles that consist of polymers, metal, or lipids without any delivery enhancing or targeting moiety to guide their transport are sometimes referred to as conventional NPs. These were the first ones to be used in AD nano-therapeutics.

### Polymer-Based Delivery System

Polymeric NPs have been used extensively for AD drug delivery to enhance the efficacy of already available drugs.

#### Chitosan NPs

Chitosan is a biocompatible, biodegradable, and non-toxic (De Campos et al., 2001) linear polyamine polymer with reactive amine and hydroxyl surface groups (Dutta et al., 2004). Owing to the presence of reactive sites, this harmless polymer has chameleon-like characteristics. By virtue of these surface groups, it can have various modifiable properties like **(i)** adjustable drug-loading capacity_ENREF_3, **(ii)** hydrophilic–lipophilic balance (HLB) value, **(iii)** pH sensitivity, **(iv)** controlled drug release, **(v)** targeting ability, and **(vi)** surface adsorption (Shu and Zhu, 2000; Liang et al., 2008; Wang et al., 2008).

The chitosan backbone can be chemically altered to adjust the HLB value of the resultant polymer (Agnihotri et al., 2004). It is suggested that the hydrophilic polymer-based DDR system that prolong the blood retention time may act as a parenteral sustained drug-release system. This characteristic of chitosan has been utilized in increasing the brain delivery of anti-Alzheimer drugs that can freely cross the BBB (Wilson et al., 2010). Chitosan has also been used to increase the brain-to-blood ratio of drugs with poor ability to cross BBB. This was achieved by using the muco-adhesive properties of the polymer by Fazil et al. (2012) in developing rivastigmine-loaded chitosan NP for intranasal delivery of drug to brain.

#### PLGA NPs

Poly (lactic-co-glycolic acid) forms one of the most attractive nanomaterials to synthesize NPs for Alzheimer’s treatment. Its properties of biocompatibility, biodegradability, modifiable surface properties, stealth character, and ability to develop modified and sustained drug releasing nano-depots make it a perfect nanodevice (Danhier et al., 2012).

Md et al. (2014) prepared nano-particulate DDR system for donepezil using PLGA. The formulation showed sustained release and efficient brain targeting in SD rats (Md et al., 2014). Similarly, in another study, Wang et al. (2010) showed that coenzyme Q(10)-loaded TMC surface-modified NPs (TMC/PLGA-NP) greatly improved the memory impairment in mice. Senile plaques and biochemical parameters also showed brain targeting potential of the TMC/PLGA-NPs (Wang et al., 2010).

#### Cyanoacrylate NPs

Cyanoacrylate polymer provides a biocompatible and bioerosive medium for the synthesis of targeted NPs for treatment of AD. Its modifiable surface properties provide a very good medium for development of the sustained release drug delivery vehicle with long blood circulating times (Yordanov, 2012). Wilson et al. (2008) used poly (*n*-butyl cyanoacrylate) NPs for enhanced delivery of tacrine and rivastigmine. The group developed rivastigmine- and tacrine-loaded polysorbate 80-coated poly (*n*-butyl cyanoacrylate) NPs in two different studies. It was noted that there was a 3.82- and 4-fold increase in the concentration of rivastigmine and tacrine in brain, respectively, with the nano-formulation as compared with the free drug (Wilson et al., 2008).

### Liposomal NPs for AD Drug Delivery

Liposomes are colloidal particles prepared by exposing phospholipids to the aqueous phase (Anwar et al., 2017). They facilitate loading of hydrophilic drugs into the aqueous interior of liposomes, thus preventing them from acid degradation, first pass effect, and delayed renal clearance (Smith et al., 2010).

Mutlu et al. (2011) have developed multilamillar rivastigmine-loaded liposomal NPs associated with permeability enhancer-like sodium-taurocholate or dimethyl-beta-cyclodextrin. Rivastigmine-sodium-taurocholate liposomes showed the highest apparent permeability coefficient in Madin-Darby Canine Kidney cells monolayer and the highest AChE inhibition in Balb-C type mice when administered through oral/intraperitoneal route (Mutlu et al., 2011). Similarly, Smith et al. (2010) have reported increased oral bioavailability by twofold and improved neuronal (SweAPP N2a cells) α-secretase inducing ability by 91% *in vitro* of epigallocatechin-3-gallate (EGCG) with use of nano-lipidic particles.

### Stealth NPs

**FIGURE 1:**
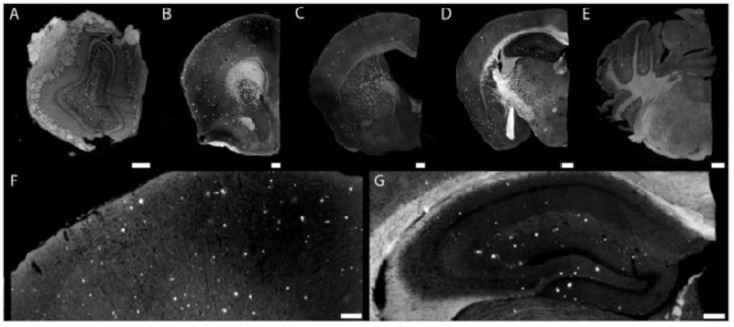
Injected targeted liposomes cross the blood–brain barrier of APP/PSEN1 mice and labeled parenchymal Ab deposits. Composite images **(A–E)** of olfactory bulb **(A)** showing plaque pathology within the granule cell layer. Nanoparticles bound to plaques at the level of the septo-striatum **(B).** Cortical pathology at the septo-diencephalic **(C)**. Hippocampal and cortical pathology **(D)** within the caudal diencephalon. Binding to cerebellar plaques (found within APP mice) within the rostral mesencephalon **(E).** An example of cortical plaque pathology **(F)** visualized at 10× magnification. Hippocampal plaque pathology **(G)** is similar to previously reported studies. Scale bar = 100 mm (adopted with permission from Tanifum et al., 2012).

As BBB favors delivery of lipophilic, neutral, and small-sized particles, most of the early NPs employed for brain delivery of therapeutic agents are hydrophobic in nature (Sahni et al., 2011). Conventional NPs overcome a number of hindrance factors associated with conventional oral drug delivery such as acid degradation, first pass effect, and low brain-to-blood ratio (Di Stefano et al., 2011). The phenomenon of surface opsonization and subsequent removal from the bloodstream through reticuloendothelial system and Kupffer cells in liver and spleen leads to substantial decrease in the systemic and local therapeutic drug concentration. In the blood, hydrophobic and charged NPs undergo rapid surface opsonization within seconds to minutes as compared to hydrophilic or neutral NPs. This surface property is used to prepare the so-called “stealth” NPs with long blood-circulating times by coating them with specific polymer. Typical characteristics of polymer used to induce stealth character to NPs include flexibility and hydrophilicity. Several polymers that are used for such purposes in the brain DDR system include natural; chitosan, semisynthetic; polyethylene glycol (PEG), poly vinyl alcohol (PVA), polyvinylpyrrolidone (PVP), PEG-based copolymer; like poloxamer, poloxamines, and polysorbates (Salmaso and Caliceti, 2013). Tanifum et al. (2012) synthesized stealth liposomal NPs incorporated with Aβ-targeting fluorescent lipid conjugate, 1,2-distearoyl-*sn*-glycero-3-phosphoethanolamine-*N*-[methoxy-XO4-(polyethylene glycol-3400)] sodium salt (DSPE-PEG_3400_-XO4), where methoxy-XO4 serves as targeting and fluorescent moiety. When administered intravenously, nano-conjugate systems showed long circulating time with high efficiency for Aβ plaques in brain sections of APP/PSEN1 transgenic mice (Tanifum et al., 2012). Figure 1 shows the injected-targeted liposomes crossing the BBB of APP/PSEN1 mice and labeled parenchymal Ab deposits.

### Nasal Route for Enhanced Delivery Across BBB

Many studies have used the nasal route for enhanced delivery of drugs across BBB because increased permeability of the nasal epithelia to drugs due to tight junction opening between apical cells provides a better route for increased drug delivery (Pardeshi and Belgamwar, 2013). It was noted that the brain delivery of drug through the nasal route is affected by the size, surface charge, and hydrophilic–lipophilic balance value of the NPs. The NPs with a size of less than 20 nm are proposed to follow the paracellular transport as compared with NPs of size 100–200 nm, which have been proposed to follow the transcellular route of drug transport. Similarly, particles with a dominant hydrophilic character follow the aqueous paracellular route of transport. Moreover, increase in cationic charge over the chitosan NPs showed increase in muco-adhesion to the nasal mucous and, hence, increased delivery across BBB (Mistry et al., 2009).

This attribute was used by Zhang C. et al. (2014) where they reported the synthesis of *Solanum tuberosum* lectin-modified polyethylene glycol-polylactide-polyglycolide NPs for enhanced intranasal delivery of basic fibroblast growth factor. The study showed 1.7-fold higher concentration of drug when administered intranasally as compared with intravenously as determined by the area under concentration–time graph of the brain based on the radioiodine isotopic method. Moreover, the neuroprotective effect of the intranasal preparation was higher as compared with the intravenous preparation as determined by the mirror water maze task in AD rats, indicating that the system is a good brain DDR system for peptide- and protein-based drugs. Similarly, in another study, Arumugam et al. (2008) showed that intranasal delivery of rivastigmine-loaded multilamellar liposomes results in higher concentration and longer half-life in the brain of Wister rats in comparison to intranasal (non- liposomal) and oral preparation.

### Targeted NPs for Delivery Across BBB

The advent of new strategies such as targeting agents, specific cell receptor ligands, and monoclonal antibodies have shifted the paradigm from non-specific neurotransmitter-based symptomatic treatment toward more personalized and targeted therapeutics that aim to disrupt unique biochemical processes necessary for plaque formation and its development (Bhaskar et al., 2010). Many researchers have explored the possibility of limiting the off-site binding and enhancing drug delivery of NPs across BBB by employing a targeting agent for specific BBB and Aβ receptors (Ulbrich et al., 2009). These agents include, but are not limited to apolipoprotein-E receptor-binding peptide and mAbs directed to transferrin and insulin receptors for BBB targeting and anti-amyloid beta antibody against Aβ_42_ for Aβ plaques targeting (Rocha, 2013). Similarly, targeting agents directed toward tau protein and GSH are also under various clinical trials.

In an *in vitro* study, Canovi et al. (2011) showed that nano-liposomes decorated with an anti-Aβ monoclonal antibody (Aβ-MAb) have a strong potential as vector for targeting Aβ_1-42_ peptides *in vitro* and plaque deposits in postmortem AD tissue. In addition to antibody-based targeting agents, several types of amyloid-specific cell receptor ligands are employed for targeted delivery of drug carriers across BBB. Gobbi et al. (2010) demonstrated in an *in vitro* immune-staining study that phosphatidic acid (PA)- and cardiolipin (CL)-decorated liposomes successfully interacted with Aβ_1-42_ aggregates, indicating that these liposomes are a potential vector for targeted delivery of anti-amyloid agents. In another similar study, Markoutsa et al. (2012) reported synthesis of dual-targeting liposomal NPs of two types: anti-Aβ–MAb (Aβ–MAb)-decorated immune-liposomes (LIP) and dually decorated LIP (dd-LIP) with OX-26 (directed toward transferrin receptor) and Aβ–MAb using biotin–STREP–biotin method. Both types of NPs showed an increase in binding (and transport) across human brain endothelial hCMEC/D3 cells model of BBB, indicating the potential of the targeting agent in increasing the binding and transport of NPs across BBB. The dd-LIP showed lysosomal uptake and it was proposed that the transport was receptor mediated, but validation of permeability across BBB needs further *in vivo*-based studies (Markoutsa et al., 2011, 2012). The group also reported, in another study, the synthesis of mono and dually decorated LIP tagged with anti-transferrin mAb alone and in combination with peptide derivative of apolipoprotein E3 (APOE) to target the low-density lipoprotein receptor-related protein (LPR), respectively. The PEG–LIP were taken as control (Markoutsa et al., 2014). *In vitro* studies in hCMEC/D3 cell monolayer showed additive effect of dual targeting in increasing the delivery across the monolayer, but *in vivo* studies in FVB mice indicated increased delivery of dual-LIP across BBB in comparison to control, but not to mono-LIP. This variable response in *in vitro* and *in vivo* studies was attributed to the presence of serum proteins. It was suggested that use of serum proteins in *in vitro* tests will be a predictive tool for targeting ability.

Moreover, benefits of the double-targeting approach were also developed for bifunctional liposomes tagged with apolipoprotein-E receptor-binding peptide for BBB targeting and with PA for Aβ binding (Balducci et al., 2014). The study in APP/presenilin 1 transgenic mice showed that the formulation successfully reduced brain Aβ burden and ameliorated memory impairment.

### Diagnostic NPs for AD

As stated earlier, the inability to pinpoint the basic pathological event or maker of AD usually results in delayed diagnosis and treatment (Ayaz et al., 2017c). Many NPs have been checked for the delivery of diagnostic machinery across the BBB. This should ideally consist of unique fluorescent active or magnetic nanomaterial that can be detected non-invasively, packaged into biocompatible nano-vesicles decorated with a detecting ligand specific to early stage biomarkers of AD (Giljohann and Mirkin, 2009; Azria et al., 2017).

The fact is that radiolabeled Aβ_40_ peptide binds reversibly and specifically to diffuse and neuritic plaques and cerebrovascular amyloid in brain tissue obtained from AD patient. Radiolabeled Aβ_40_ peptide can be imaged *in vivo* non-invasively from the basis for its use in diagnostic and theranostic modalities related to AD. In order to improve its delivery across the BBB, targeting agents such as mAb, fusion proteins, and natural diamines are linked to it (Rocha, 2013). Some of these targeting strategies have been used in combination of NPs.

Yin et al. (2015) synthesized dimercaptosuccinic acid-coated magnetic NPs (MNPs) tagged with anti-amyloid antibodies. They showed that the synthesized NPs were biocompatible and biologically active with the potential of being used for early detection of AD (Yin et al., 2015). Similarly, in another study, Tanifum et al. (2016) reported the synthesis of amyloid fibril-binding ligand ET6-21. The ligand was used to prepare amyloid-targeted liposomes, tagged with Gd chelates and indocyanine green for visualization by magnetic resonance imaging (MRI) and infrared microscopy, respectively. The study showed successful binding of the particles to the plaques after intravenous administration in transgenic 2576 and APP (mutant APP) mouse models of AD. The study indicated the potential for development of MRI imaging agents for detection of amyloid plaques. Similarly, another group synthesized curcumin-loaded super paramagnetic iron oxide NPs of <100 nm for targeting of amyloid plaques (Cheng et al., 2015). The synthesized NPs were successful in differentiating the transgenic 2576 mouse brains having Aβ plaques from non-transgenic mouse models, indicating their potential as a diagnostic agent in AD.

Moreover, recent studies showed that Aβ oligomer instigate the early stage of memory loss and thus, is a better candidate as a biomarker of early-stage identification of AD. Viola et al. (2015) synthesized Aβ oligomer-targeted molecular MRI probes by attaching AβOs-specific monoclonal antibody (NU4) to the nitro-dopamine and polyethylene glycol stabilized 12–16 nm magnetite NPs (Viola et al., 2015). The study showed significant clinical potential of the AβO–MRI probe for identification of synapto-toxic oligomers in 5xFAD mouse model following intranasal delivery.

### Theranostic NPs for Delivery Across BBB

Many preclinical studies have established the role of NPs as carriers and use of specific surface ligands for targeting the NP for AD separately. This leads to the development of multimodal preparations having characteristics of drug carriers tagged with targeting moieties and diagnostic agents. These magical NPs with all-in-one characteristics are the next-generation particles termed as “theranostic agent.”

Papadia et al. (2017) synthesized multifunctional liposomal NPs decorated with curcumin–lipid ligand (TREG) with affinity toward amyloid species along with ligands to target the transferrin and the LDL receptors of the BBB. The study showed successful targeting of brain and inhibition of amyloid peptide aggregation (Papadia et al., 2017). Similarly, Rotman et al. (2015) have also developed two liposomal formulations based on 1,2-dimyristoyl-sn-glycero-3-phosphocholine and egg-yolk PC encapsulating indium-111-labeled Aβ-binding llama single-domain antibody fragments (VHH-pa2H) and were coated with glutathione–PEG. The animal study in APP_swe_/PS1dE9 double transgenic mice showed the highest standard uptake in brain with GSH–PEG EYPC, indicating the theranostic potential of the prepared formulation (Rotman et al., 2015).

### Green-Synthesized NPs as Future AD Theranostic Agents

Recent developments at the interface of green chemistry and nanotechnology indicate significant potential in the biomedical sciences from theranostic perspectives (Ovais et al., 2016, 2017b, 2018a,d; Barabadi et al., 2017b; Khalil et al., 2017a). However, their potential is largely untapped in case of the neurodegenerative diseases like Alzheimer’s. The use of other synthesis methods (physical and chemical) is often discouraged as they possess certain inherent disadvantages such as toxicity and cost (Khalil et al., 2017b; Ovais et al., 2017a). Moreover, in a recent work, it was reported that some chemicals used during the synthesis of NPs through a chemical route can remain attached on the surface of the NPs and, therefore, could not be used for biomedical application. Therefore, tremendous interest has been growing over the last decade to fabricate materials of interest using a green process (Emmanuel et al., 2017). Usually, the green chemistry-based approach includes the use of medicinal plants or pure phytochemicals that have potential medicinal value. These phytochemicals are involved in the chelating as well as stabilizing of NPs (Barabadi et al., 2017a; Ovais et al., 2017b, 2018c; Khalil et al., 2018).

**FIGURE 2:**
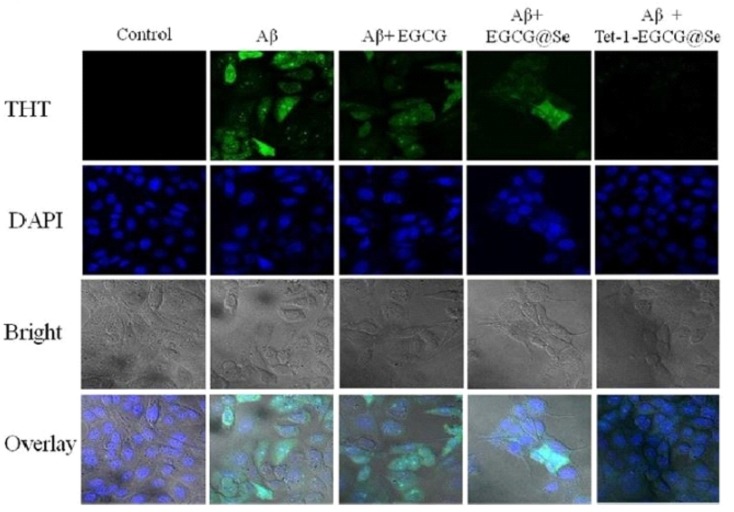
Tet-1-EGCG@Se preventing Aβ aggregation in PC12 cells. The presence of intracellular Aβ fibrils was evaluated by ThT staining in PC12 cells in the absence and presence of EGCG, EGCG@Se, and Tet-1-EGCG@Se. The cells were pretreated with an Aβ monomer for 6 h to allow access of Aβ to the cytoplasm, sequentially incubated with EGCG, EGCG@Se, or Tet-1-EGCG@Se for an additional 48 h, and visualized under a fluorescence microscope (adopted with permission from Zhang J. et al., 2014).

**FIGURE 3:**
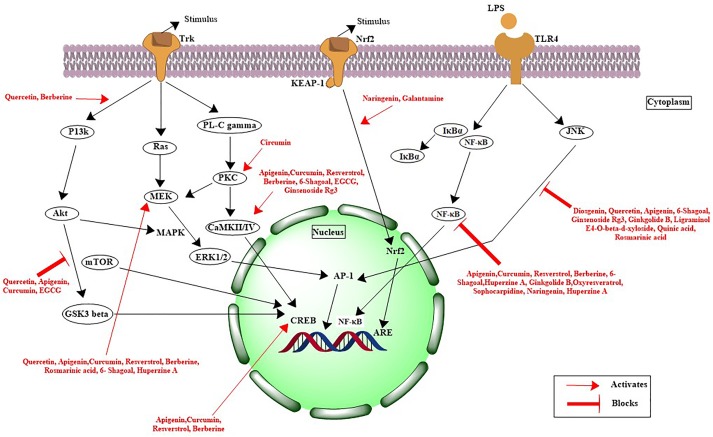
Mechanistic pathways for natural products involved in neuroprotection against Alzheimer’s disease.

Recently, Suganthy et al. (2017) reported the neuroprotective nature of biogenic gold NPs synthesized through *Terminalia arjuna*. Their results indicated the significant neuroprotective nature and biocompatibility of biogenic gold NPs. The biogenic gold NPs successfully inhibited the ChE enzymes, reduced the Aβ fibrillation process, and also destabilized the mature fibrils at very low concentrations. The functionalization of chemically synthesized gold NPs with trehalose has significantly improved the inhibition of protein aggregation as well as the mature fibril disintegration and possesses the potential to be used in photothermal therapies (Mandal et al., 2017). Functionalization of bioinspired gold NPs with such antiamyloidogenic molecules can be a fruitful strategy for further enhancing their neuroprotective nature. Another research contribution directed toward the neuroprotective behavior included the biogenic platinum NPs biosynthesized through *Bacopa monnieri*. The authors concluded that the neuroprotective potential of biogenic platinum nanoparticles is mediated via inhibition of free radicals generation and antioxidant effects (Nellore et al., 2013). Plant phytochemical-based functionalization of NPs has yielded amazing results. Recently, polyphenol-based functionalization of selenium NPs was achieved. In the research, nanoscaled selenium was coated with EGCG; a polyphenol found in the tea. The EGCG is known for its neuroprotective potential and possesses the ability to inhibit various amyloid-forming proteins such as amyloid beta, transthyretin, α-synuclein, and huntingtin, which are involved in the progression of AD. The EGCG-stabilized Se NPs were further coated with Tet-1 proteins, having strong neuronal affinity. The Tet1-Se@EGCH nanosystem induced effective inhibition of the fibrillation process of Aβ and also disintegrated the already-matured fibrils as shown in Figure 2. In addition, the indicated nanosystem was found to inhibit the DNA fragmentation and ROS generation while found to be effective at very low concentrations (Zhang J. et al., 2014). There is potential to replace the EGCG with other plant-based chemical entities. Curcumin or curcumin-based derivatives have yielded impressive properties to halt the disease progression of Alzheimer’s. Curcumin in conjunction with the benzothiazolinone possesses strong affinity for binding to amyloid and, hence, can be used to deliver curcumin or other natural products with anti-Alzheimer potential as summarized in Figure 3 (Megill et al., 2013; Fang et al., 2014).

## Conclusion and Future Prospects

Recent research on phyto and nanomedicines represents a potential area of interest in the treatment of AD. Numerous phytochemicals have proven promising results in AD therapy, while with the emerging feat of tailored nanomedicines, the effectiveness can be increased several folds by efficient delivery of the active ingredients to the target sites. Moreover, a relatively untapped area of research at the interface of medicinal plants and nanotechnology is the biogenic synthesis of metal NPs for AD treatment.

The occurrence of metal and metal-based NPs in the brain can be toxic as well as beneficial. The NPs like TiO_2_, ZnO, Ag, and Au NPs etc. can induce oxidative damage in the brain. However, selenium NPs are reported to have an antioxidant role in the brain. Largely, these mentioned NPs were synthesized through chemistry-based methods. The green chemistry-based methods can alter the properties of these NPs. The science of biogenic NPs is still in the nascent phase, and theranostic NPs of variable and interesting features can be produced to alleviate the menace of AD.

## Author Contributions

MO, NZ, and IA conceived the idea and drafted the manuscript. AK, AR, MA, AS, FU, and ZS contributed in manuscript drafting, language correction, and technical corrections. All the authors read and approved the manuscript.

## Conflict of Interest Statement

The authors declare that the research was conducted in the absence of any commercial or financial relationships that could be construed as a potential conflict of interest. The reviewer EL and handling Editor declared their shared affiliation at the time of the review.
